# Energy-free machine learning force field for aluminum

**DOI:** 10.1038/s41598-017-08455-3

**Published:** 2017-08-17

**Authors:** Ivan Kruglov, Oleg Sergeev, Alexey Yanilkin, Artem R. Oganov

**Affiliations:** 1 Moscow Institute of Physics and Technology, Dolgoprudny, 141700 Moscow Region Russian Federation; 2 Dukhov Research Institute of Automatics (VNIIA), Moscow, 127055 Russian Federation; 3 Skolkovo Institute of Science and Technology, Skolkovo Innovation Center, Moscow, 143026 Russian Federation; 4 Stony Brook University, Stony Brook, New York 11794-2100 USA

## Abstract

We used the machine learning technique of Li *et al*. (PRL 114, 2015) for molecular dynamics simulations. Atomic configurations were described by feature matrix based on internal vectors, and linear regression was used as a learning technique. We implemented this approach in the LAMMPS code. The method was applied to crystalline and liquid aluminum and uranium at different temperatures and densities, and showed the highest accuracy among different published potentials. Phonon density of states, entropy and melting temperature of aluminum were calculated using this machine learning potential. The results are in excellent agreement with experimental data and results of full ab initio calculations.

## Introduction

Computational chemistry tools, and more specifically molecular modeling, play an increasingly important role. Many properties of various physical systems, their energies and forces acting on atoms may be studied using parametrized explicit functions, called force fields or interatomic potentials. For example, dislocation movement, shock-wave response and diffusion of defects in metals are often modeled with embedded atom method^1^ or angular-dependent potentials^2^. Processes in proteins and lipids are simulated with AMBER^3^ and CHARMM (for example, see ref. 4) force fields. Chemical processes such as catalysis, polymerization and isomerization are studied with the ReaxFF^5^ potential. There is a wide range of other techniques and applications. Classical interatomic potentials are in general much faster and scale better than *ab initio* methods. However, they cannot precisely reproduce quantum-mechanical forces and have limited transferability.

The development of new methods capable of better representing potential energy surfaces is of great interest to computational chemistry and materials science. Machine learning (ML) approaches have the advantage of being more flexible and capable of reproducing reference data more accurately than traditional force fields. ML force field could be both potentials in the traditional sense that for a given atomic configuration calculate the energy of a system, or alternative methods that directly reproduce the forces. Their common feature is that there is no predefined functional form for interatomic interactions, but instead energies or forces are calculated as a (finite) sum of basis functions that may in principle be capable of reproducing complex actual functions in configurational space. One of the first ML potentials was proposed in^6, 7^, where the interaction of different gases with surface was simulated using neural networks (NN). Later these approaches were improved and applied in refs 8 and 9. In these works the interaction potential was given by the coefficients of a neural network. Symmetry functions were used to describe atomic configurations, including radial and angular distributions of atoms. Using Parrinello-Behler ML potential, solid and liquid sodium were described in refs 10 and 11, and in combination with metadynamics, mechanisms of phase transitions in silicon were studied^12^. This method was then extended to systems consisting of several types of atoms^13^ and applied to water^14^, methanol^13^ and zinc oxide^15^.

Another ML technique for interatomic potentials is GAP (Gaussian approximation potential)^16^, which is based on the bispectrum decomposition and Gaussian regression. The GAP potentials were developed to describe tungsten and its defects^17^, solid and liquid water^18^ and amorphous carbon^19^. Similar to GAP is SNAP (Spectral Neighbor Analysis Potential)^20^, where the dependence of energy on bispectrum components is described with linear regression instead of the Gaussian one. Due to its simplicity and speed, linear regression algorithm was used in ref. 21, where the authors developed a new machine learning potential that can describe atomic forces and energies using invariant polynomials as descriptors (MTP). Active learning method based on D-optimality criterion appeared to be highly efficient for on-the-fly learning^22^. Besides the above mentioned interatomic potentials based on machine learning, there are many others^23–25^, the latest reviews can be found in refs 26 and 27.

In the molecular dynamics (MD) method, the behavior of the system is completely determined by forces acting on atoms and by initial conditions. Besides, there is only one value of energy per configuration, while there are 3*N* force components (*N* being the number of atoms), so one has much richer reference datasets of forces rather than energies. A ML model can be trained on both energies and forces, but we were curious to exploit a recent simple approach based solely on forces^28^. We still call it an interatomic potential, even though the energy is not used in this method, and we test this energy-free method on quantities that depend both on the forces (phonons, entropies) and energies (melting temperature).

Theoretical methods based on density functional theory (DFT) are predictive, but often prohibitively expensive. One way to calculate the free energy is the thermodynamic integration method^29, 30^. The complicated part of that approach is to build a reference system which would have properties similar to the real system. Another way is to integrate the phonon density of states, which can be calculated using the frozen phonon method^31^. The effects of anharmonicity can be taken into account by the self-consistent phonons method^32^ or by perturbative corrections^33^. The former approach accounts for finite displacements of atoms, while the latter accounts for finite lifetime of phonons.

In principle, the phonon density of states can be calculated using molecular dynamics at finite temperature as a Fourier transform of the velocity autocorrelation function (VACF)^34^. This allows to take into account naturally the displacements of atoms and the finite lifetime of phonons. However, this approach requires the use of large systems and, therefore, is very expensive in the context of quantum molecular dynamics calculations. Instead, classical MD would be a practical approach if an accurate interatomic potential were available.

In this work we use an approach for atomic forces reconstruction which is similar to the one proposed in ref. 28. We also apply feature matrix as a descriptor for local atomic configuration, and linear regression for fitting the relationship between the descriptor and force. Crystalline and liquid phases of aluminum and uranium were investigated. Al was chosen because there are a plenty of experimental data for its properties (density, melting temperature and so on), and many interatomic potentials were also developed for it. On the contrary, the published interatomic potentials for U give large errors in forces, and the phase diagram of U is still unknown. In this work we will examine crystalline *α*-U (stable up to 235 GPa^35^) and liquid uranium.

## Method

Following^28^, for each atom a set of *k* internal vectors is defined as

1 $${{\bf{V}}}_{i}=\sum _{q=1}^{{N}_{neigh}}{\hat{{\bf{r}}}}_{q}exp[-{(\frac{{r}_{q}}{{r}_{cut}(i)})}^{p(i)}],\quad i=1\ldots k,$$

where *N*_*neigh*_ is the number of neighbouring atoms, *r*_*cut*_ and *p*–constants that must be chosen optimally, *r*_*q*_ = ||**r**_*q*_||, ${\hat{{\bf{r}}}}_{q}={{\bf{r}}}_{q}/{r}_{q}$. For convenience a set of *k* internal vectors and *k* collinear with them unit vectors can be written in the form of two matrices *V* and *A*:

2 $$V=(\begin{array}{c}-{{\bf{V}}}_{1}-\\ \ldots \\ -{{\bf{V}}}_{k}-\end{array}),\quad A=(\begin{array}{c}-{\hat{{\bf{V}}}}_{1}-\\ \ldots \\ -{\hat{{\bf{V}}}}_{k}-\end{array})$$

Then for each atom the matrix *X* is built, ${X}_{ij}={{\bf{V}}}_{i}\cdot {\hat{{\bf{V}}}}_{j}={(V{A}^{T})}_{ij}$. This *X* here is the feature matrix. Each *X* matrix corresponds to a vector ${\mathcal F}$, which consists of projections of **F** on ${\hat{{\bf{V}}}}_{i}$: ${\mathcal F} =A{\bf{F}}$ (**F** = *A* ^+^ ${\mathcal F}$, where *A* ^+^ is the pseudoinverse matrix for *A*). To establish the relationship between *X* and ${\mathcal F}$, we use linear regression. In other words, we find the coefficients vector Θ in the equation *X* ^*T*^Θ = ${\mathcal F}$. The components of Θ are free parameters in the method. When determined, Θ, together with the set of (*r*_*cut*_; *p*) pairs and with the radius ${R}_{cut}^{global}$ of the sphere in which the neighbors are counted, form the interatomic potential for MD.

We implemented this potential in the LAMMPS^36^ code. A particular feature of the method is that it does not compute the potential energy. This, however, does not affect the dynamics. The implementation of the potential is parallelized using LAMMPS domain decomposition.

We developed several parameterizations of our ML potential for aluminum and uranium. Trajectories for training sets were taken from first-principles molecular dynamics calculations made with VASP^37^ for different densities and temperatures. Each trajectory was calculated with a time step of 1 fs for about 1 ps.

### Force field parametrization

**Figure 1:**
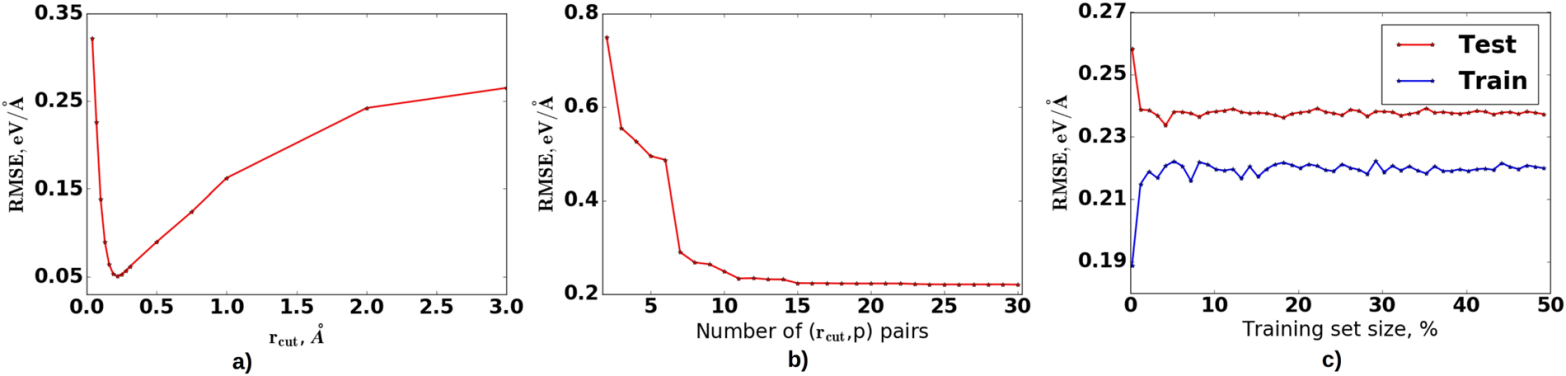
The optimal choice of number of parameters pairs and training set size. (**a**) The RMSE dependence on the value of *r*_*cut*_ at a given *p* = 1, (**b**) the relation between RMSE on test set for *α*-U and number of parameters pairs, (**c**) learning curves when randomly selected structures are in the training set.

As we mentioned above, dynamics of the system are mostly defined by forces acting on them and by initial conditions. So, the small difference between ab initio and predicted forces (root mean square error, RMSE) was considered as the main quality criterion for constructed potentials. In order to parameterize any potential, the particular pairs of values (*r*_*cut*_, *p*) were selected manually. First we fixed *p* = 1 and plotted RMSE dependence on the value of *r*_*cut*_. Therefore, the starting pair of parameters was defined by the minimum of the RMSE value on this plot. The subsequent values of constants were taken with the step of 0.3 in *r*_*cut*_ and *p* units. For example, we found that for aluminum at zero pressure and 300 K the optimal value of *r*_*cut*_ was 0.22 Å at *p* = 1, RMSE = 0.043 eV/Å (Fig. 1a). For uranium this minimum is very broad. For this case *p* was taken in the range from 1 to 3, and *r*_*cut*_–from ${R}_{cut}^{global}$ to 1. We note that *r*_*cut*_ = 0.22 Å is similar to the exponent *β* = 0.25 Å in the Morse potential (which is just a sum of two exponents). The model used here can be thought of as generalized Morse potential with many-body effects.

The main parameters which should be optimized for ML potentials are not only the exact values of *r*_*cut*_ and *p* pairs, but also the number of such pairs and training set size. Since Al even with one optimally selected pair of parameters could be relatively well described (see Fig. 1a), all the main features of ML potential will be considered with reference to uranium *α*-phase (at zero pressure and 1000 K).

First, we established the optimal number of pairs (*r*_*cut*_, *p*) (Fig. 1b). To do this the training set was chosen to be 20% of the whole dataset. The figure shows that the minimum value of error could be reached using 15 pairs. But for the molecular dynamics runs feature vector calculation time (which linearly increases with number of parameters) plays a crucial role, so for further calculations the number of (*r*_*cut*_, *p*) pairs was taken as a compromise between calculation time and RMSE. Figure 1b shows that the optimal number of pairs equals to 11, and this is common for almost all ML potentials considered here.

Second, after the optimum number of (*r*_*cut*_, *p*) pairs was defined, we studied the RMSE dependence on the training set size. We randomly chose structures from the first 50% steps of MD trajectory and put them in the training set (Fig. 1c) (for the test set we always left the last 50%). There exist smarter strategies such as active learning^22^ and evaluation of distance from a given structure to other structures for its further consideration as a new point in the training set^28^. However, using our approach, convergence in error was achieved even when there were 10% of all structures in the training set. Normally, for confidence, we took 20% of the structures for training. Since database of structures normally consisted of only 1 ps MD run, we can not affirm that constructed potentials will not be overfitted. In this case in machine learning the most common practice is to use regularization terms in the loss function. It penalizes the model for a high values of parameters (which is a typical sign of overfitting). More specifically, we added $\lambda {\sum }_{k=1}^{{N}_{pairs}}{{\rm{\Theta }}}^{2}$ term, where *λ* is a free parameter.

**Figure 2:**
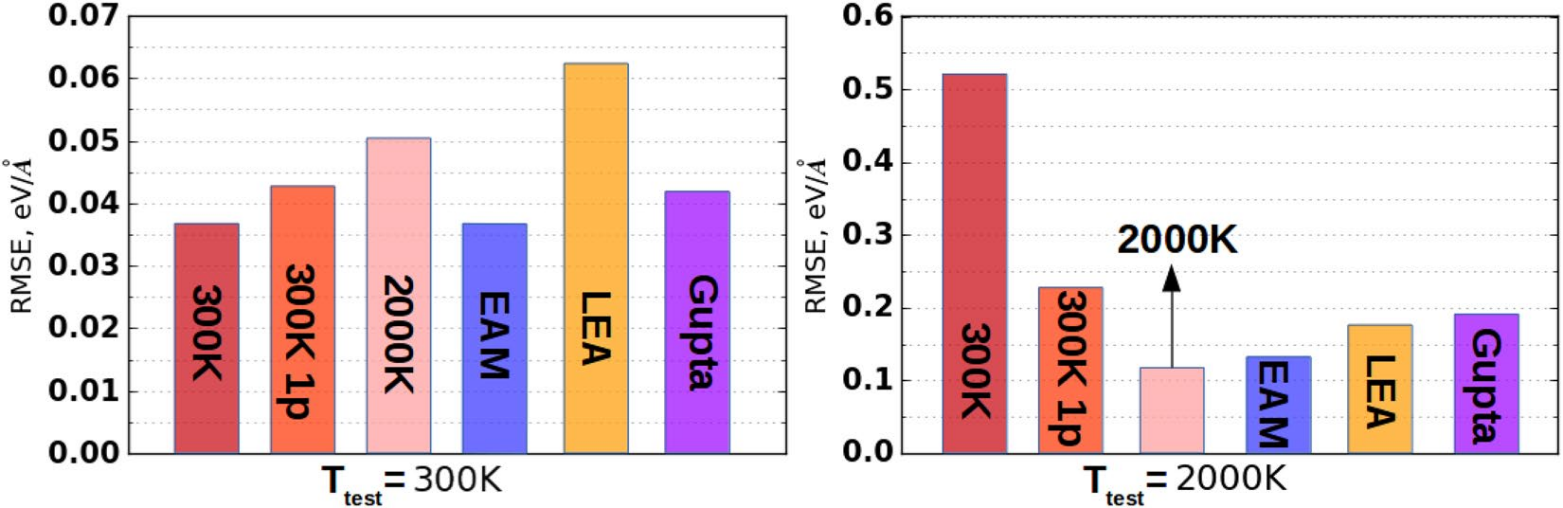
Comparison of different potentials for Al at 300 K (left) and 2000 K (right), where “300 K”–our potential trained at 300 K with 11 pairs of parameters, “300 K 1p”–our potential trained at 300 K with 1 pair of parameters, “EAM”–EAM potential trained on the same training set, “2000 K”–our potential trained at 2000 K with 11 pairs of parameters, “LEA”–from^38^, “Gupta”–from^39^.

We compared the accuracy given by our ML potentials for Al and U and by different published embedded atom method (EAM) potentials. We also compared our potentials with the EAM potential constructed by us using force matching technique based on the same training set. The latter type of potentials was included for a more fair comparison. For Al we studied fcc phase at 300 K and liquid phase at 2000 K (Fig. 2). At 300 K our potential with 11 pairs of parameters gave the same accuracy as EAM potential made using force matching. Yet these errors were lower than the ones given by^38, 39^ potentials. Even our potential trained with one pair of parameters had accuracy higher than potentials in refs 38 and 39. Moreover, the potential parameterized at 2000 K accurately predicts forces for structures at 300 K. The lowest RMSE for test MD trajectory corresponding to 2000 K was reached using our ML potential with 11 pairs of parameters.

**Figure 3:**
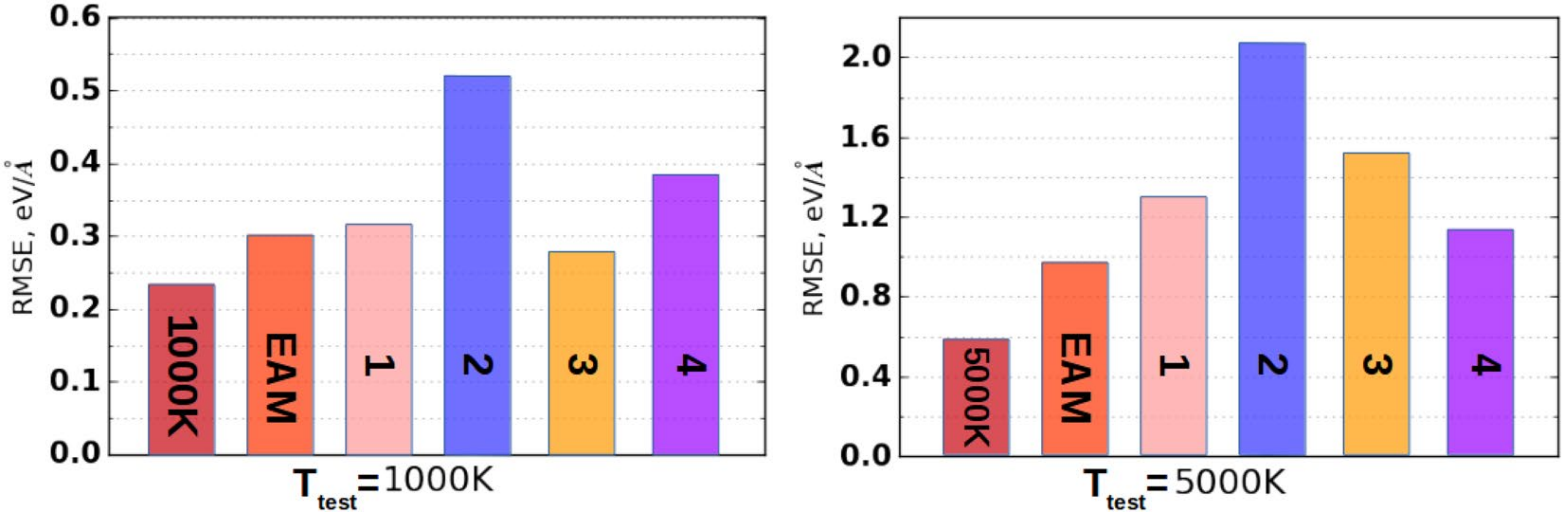
Comparison of different potentials for *α*-U at 1000 K (left) and bct U at 5000 K (right), where “1000 K” and “5000 K”–our potential trained at 1000 K and 5000 K with 11 pair of parameters respectively, “EAM”–EAM potential trained on the same training set, 1–from^40^, 2–from^41^, 3–from^42^, 4–from^43^.

Considering uranium, we tested different potentials for *α*-phase at 0 GPa and 1000 K (stable solid phase) and liquid phase at 300 GPa and 5000 K (Fig. 3). For both *α*- and liquid phases our ML potential trained with 11 pairs of parameters gave the highest accuracy among all considered potentials. In our opinion it can be used to build the phase diagram of uranium.

In the next part, we apply our ML potential to the calculation of thermodynamic properties of Al.

### Thermodynamic quantities and phase transitions

#### Phonon density of states and entropy

MD simulations were performed in a 20 × 20 × 20 supercell with periodic boundary conditions in all directions. The interactions between atoms were described with the ML potential derived in this work.

The system was equilibrated using MD in the NVT ensemble for 4 ps. After that we performed calculations of VACF in the NVE ensemble for another 4 ps (since only conservative Newton’s equations of motion are solved, we indeed have the NVE-ensemble). The characteristic time of VACF attenuation in the considered systems is about 1 ps. The phonon density of states (PDOS) was calculated using the formula

3 $$g(\nu )=4\cdot {\int }_{0}^{\infty }cos\mathrm{(2}\pi \nu t)\frac{\langle \overline{v\mathrm{(0)}v(t)}\rangle }{\langle {\overline{v\mathrm{(0)}}}^{2}\rangle }dt$$

where *ν* is the vibrational frequency, and the average is taken over all atoms. The system must be large, if accurate *g*(*ν*) is needed (e.g., 4 atoms in the unit cell ×20 × 20 × 20 = 32000 atoms in our calculations), so one cannot use ab initio molecular dynamics even though the necessary physical calculation time is rather short.

**Figure 4:**
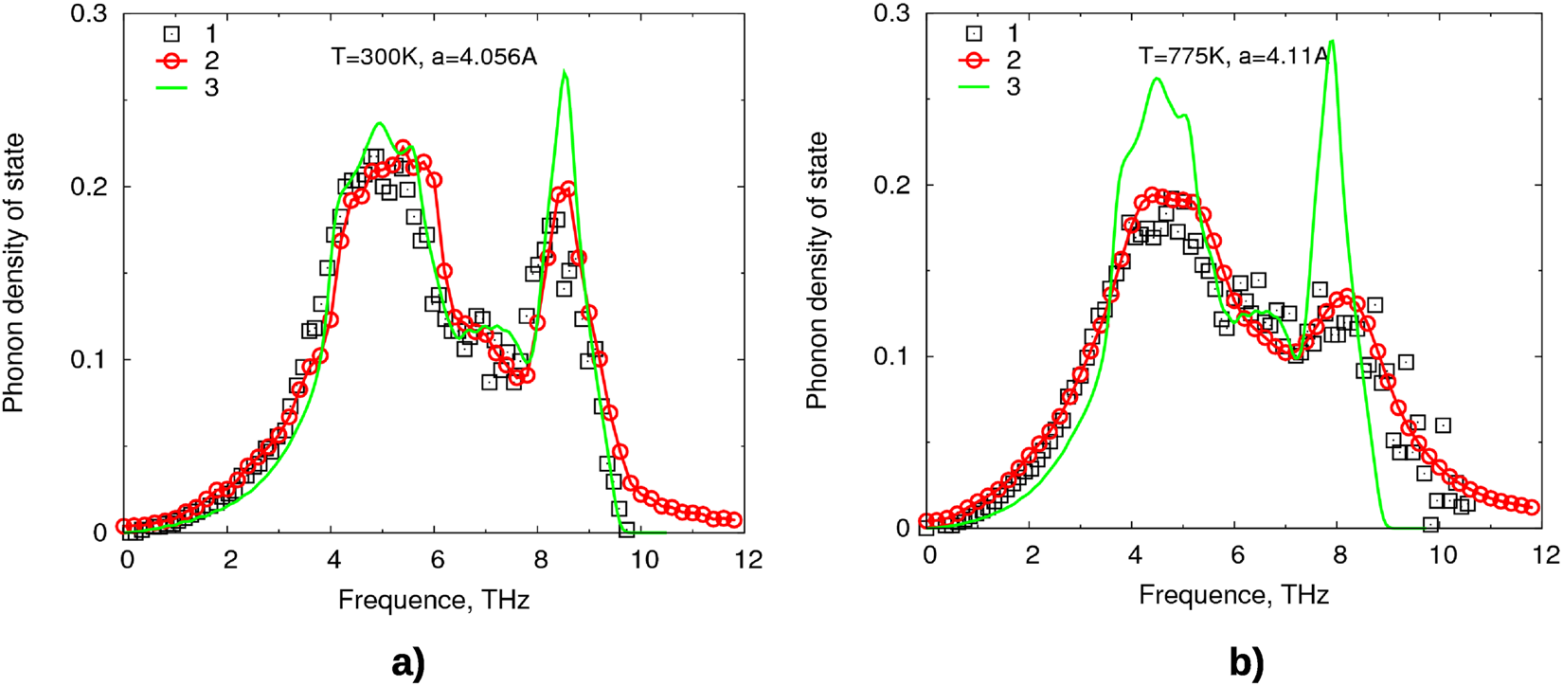
Phonon density of states at (**a**) 300 K and (**b**) 775 K: 1 - experimental data from^44^, 2 - calculation via MD with our ML potential, 3 - calculation via frozen phonon method (using DFT).

Figure 4 shows two examples of the calculation of the phonon density of states. Positions, widths and heights of peaks are in good agreement with the experimental data from inelastic neutron scattering^44^. The results differ substantially from calculations made with the frozen phonon method. In the frozen phonon method, a purely harmonic PDOS is obtained, neglecting anharmonicity and finite lifetime of phonons. The finite displacements can be accounted for using the self-consistent phonon method suggested in ref. 32, and the broadening due to finite lifetimes can be calculated from phonon-phonon interaction (taken from perturbation theory)^31^. In the approach used here, these two effects appear naturally from the movement and interaction of atoms at finite temperature. We also checked, that the behavior of PDOS does not really change, if PDOS is calculated using first 2 ps from MD run or the latter 2 ps.

Entropy was computed using the harmonic formula:

4 $$S=3{k}_{B}{\int }_{0}^{\infty }g[(n+\mathrm{1)}\,\mathrm{ln}(n+\mathrm{1)}-n\,\mathrm{ln}\,n]d\nu $$

where *k*_*B*_ is the Boltzmann constant, *g* = *g*(*ν*) − phonon density of states, *n* = *n*(*ν*) = 1/(*exp*(*hν*/*k*_*B*_ *T*) − 1)–average density of bosons. However, the *g*(*ν*) used in Eq. 4 includes all anharmonic effects. It is known^45^ that the use of Eq. 4 in conjunction with anharmonically renormalized *g*(*ν*) yields correct entropies, including anharmonic effects to the leading order of perturbation theory.

**Figure 5:**
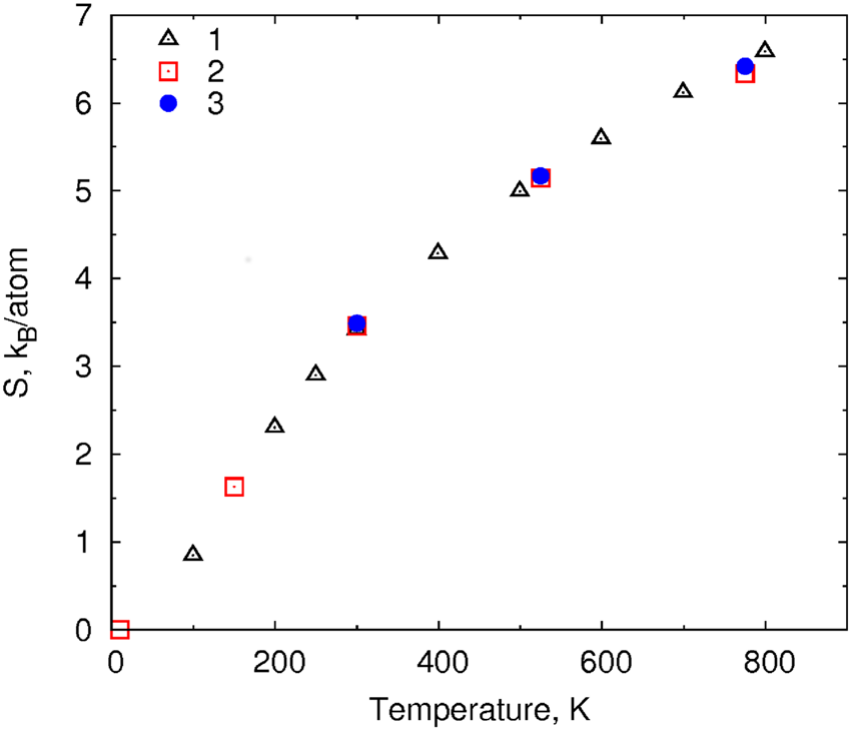
The entropy dependence on temperature: 1 - thermodynamic data from the NIST-JANAF database, 2 - calculated from the experimental PDOS^44^, 3 - calculated from MD with the ML potential.

The computed entropies are shown in Fig. 5 and in Table 1. The obtained values are in good agreement with the experimental data. The discrepancy is within 0.1 *k*_*B*_ per atom, which enables the use of this approach for the analysis of phase stability. Similar calculations were made for several ML potentials built on the same database: the maximum spread of the entropy at room temperature is within 0.03 *k*_*B*_ per atom.

**Table 1:** Experimental and calculated entropies of crystalline Al at different temperatures.

*T*, K	300	525	775
*a*, Å	4.056	4.079	4.11
*S*_*calc*_, *k*_*B*_/*atom*	3.49	5.17	6.42
*S*_*exp*_, *k*_*B*_/*atom*	3.462	5.146	6.332

### Structure of the liquid phase and the melting point

**Figure 6:**
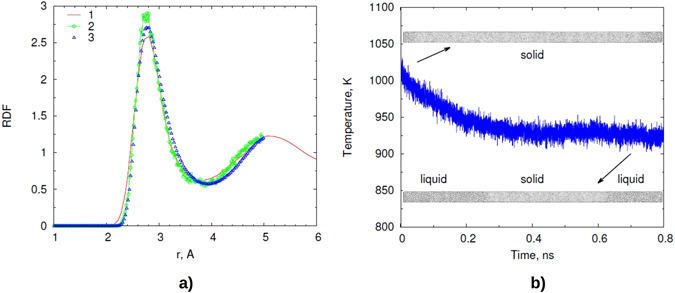
Radial distribution function and melting temperature of Al, (**a**) radial distribution function at *T* = 1023 K and *V*_*at*_ = 19.1 Å^3^: 1 - experimental data, 2 - QMD results, 3 - MD calculation with our ML potential. (**b**) The dependence of temperature on time in the calculation of the melting temperature with the modified Z method. The atomic configurations in the beginning and in the end of the calculation are also provided.

Our tests show that the constructed ML potentials can be used to reproduce the forces acting on atoms in the liquid state. For the liquid state one cannot define the phonon density of states, but the verification of the potential can be carried out on the basis of the radial distribution function (RDF). The RDF was averaged for 10 ps after equilibration (see Fig. 6a). We considered a 4000-atom supercell of Al (*V*_*at*_ = 19.1 Å^3^) at a given average temperature *T* = 1023 K. The developed potential reproduces QMD results at the same conditions and is in good agreement with experimental data. It is worth noting that almost identical results were obtained for different parameterizations made with different sets (*r*_*cut*_, *p*).

We also noted that the potential, which is parametrized on liquid configurations, also describes well the forces in crystalline configurations. Even though there are no explicitly calculated energies, a sufficiently accurate representation of the forces can enable the use of such potentials for modeling two-phase systems and for direct determination of the melting temperature. To verify this, we calculated the melting temperature using the modified Z method^46^. The system was simulated at a fixed density in the NVE ensemble. It contained 4 × 4 × 100 fcc unit cells with lattice parameter *a* = 4.16 Å. Initially the temperature was set to *T* = 2000 K, shortly after the start of the MD run it relaxed to an average temperature *T* ≈ 1000 K. After spontaneous melting, a decrease in temperature to an average value *T* ≈ 925 K was observed. The density of liquid is calculated from the density profile and corresponds to the atomic volume *V*_*liq*_ = 18.6 ± 0.1 Å^3^. The obtained atomic volume for the crystalline part *V*_*cryst*_ is 17.3 ± 0.2 Å^3^. The obtained values are in reasonable agreement with the experimental melting temperature of 933 K and the equilibrium atomic volume for liquid of 18.9 Å^3^ (ref. 47). Our results *T* ≈ 925 K and Δ*V* = *V*_*liq*_ − *V*_*cryst*_ = 1.3 Å^3^ are close to thermodynamic calculations based on DFT^30^: the melting temperature *T* = 912 K and Δ*V* = 1.35 Å^3^.

It is worth noting that pressure calculated in our QMD run was 2 ± 0.5 GPa. It is known that DFT calculations with GGA functional overestimate pressure, and in ref. 30 the pressure correction for the melting curve at normal conditions was estimated as 1.6 G Pa. Therefore, calculated pressure with correction is close to normal conditions.

## Conclusions

In this paper the machine learning technique of Li *et al*. (PRL 114, 2015) was used to reproduce the forces acting on atoms. The method is based on feature matrix description of atomic configurations and linear regression for the fitting. A number of parameterizations were obtained for Al and U at different pressures and temperatures. The comparison of our ML potential and other published potentials showed that the ML potential gives the best accuracy. Potentials constructed from liquid configurations are suitable for describing crystalline configurations as well. The verification of the proposed approach was done through comparison of the phonon density of states, entropy, radial distribution function and melting temperature with the experimental values. Phonon density of states was calculated on the basis of the velocity autocorrelation function. This approach allows one to take into account the change of vibrational frequencies and broadening of peaks. The results are in good agreement with experiment. Calculated vibrational entropy differs from experimental data by less than 0.1 *k*_*B*_ per atom. The calculated melting temperature was also shown to be in excellent agreement with experimental data and much more expensive ab initio estimates.
